# Risk Assessment of Deep Coal and Gas Outbursts Based on IQPSO-SVM

**DOI:** 10.3390/ijerph191912869

**Published:** 2022-10-08

**Authors:** Junqi Zhu, Li Yang, Xue Wang, Haotian Zheng, Mengdi Gu, Shanshan Li, Xin Fang

**Affiliations:** 1 School of Economics and Management, Anhui University of Science and Technology, Huainan 232000, China; 2 School of Safety Science and Engineering, Anhui University of Science and Technology, Huainan 232000, China

**Keywords:** deep coal mine, coal and gas outbursts, IQPSO, SVM, coal mine safety

## Abstract

Coal and gas outbursts seriously threaten the mining safety of deep coal mines. The evaluation of the risk grade of these events can effectively prevent the occurrence of safety accidents in deep coal mines. Characterized as a high-dimensional, nonlinear, and small-sample problem, a risk evaluation method for deep coal and gas outbursts based on an improved quantum particle swarm optimization support vector machine (IQPSO-SVM) was constructed by leveraging the unique advantages of a support vector machine (SVM) in solving small-sample, high-dimension, and nonlinear problems. Improved quantum particle swarm optimization (IQPSO) is used to optimize the penalty and kernel function parameters of SVM, which can solve the optimal local risk and premature convergence problems of particle swarm optimization (PSO) and quantum particle swarm optimization (QPSO) in the training process. The proposed algorithm can also balance the relationship between the global search and local search in the algorithm design to improve the parallelism, stability, robustness, global optimum, and model generalization ability of data fitting. The experimental results prove that, compared with the test results of the standard SVM, particle swarm optimization support vector machine (PSO-SVM), and quantum particle swarm optimization support vector machine (QPSO-SVM) models, IQPSO-SVM significantly improves the risk assessment accuracy of coal and gas outbursts in deep coal mines. Therefore, this study provides a new idea for the prevention of deep coal and gas outburst accidents based on risk prediction and also provides an essential reference for the scientific evaluation of other high-dimensional and nonlinear problems in other fields. This study can also provide a theoretical basis for preventing coal and gas outburst accidents in deep coal mines and help coal mining enterprises improve their safety management ability.

## 1. Introduction

Many researchers and scientists are convinced that the use of coal as an energy carrier should be phased out as soon as possible. However, as one of the primary energy sources in the world, coal will still occupy an important position in the world’s energy structure for a long time to come. With the gradual depletion of high-quality shallow coal resources, coal mines have gradually entered the stage of deep mining. Generally, “deep mining” means that the mining depth is below 600 m. Deep mining faces the objective reality of high ground temperature, high ground pressure, high gas content, and low coal seam permeability compared to shallow coal mines [1]. Research shows that the airflow temperature of the mining face increases by about 1.5 °C for every 100 m increase in mining depth [2,3]. According to Provisions on Geothermal Survey for Geological Exploration of Coal Resources, deep wells below 1000 m in China are generally in Grade I or II heat damage zones [4]. The mining depth is proportional to the original rock stress and tectonic stress. The vertical initial rock stress is 20 MPa when the mining depth is 800 m and increases to 27 MPa when the mining depth is 1000 m, which far exceeds the compressive strength of the general engineering rock mass [5]. At high temperatures and pressures in the deep coal mine, coal rocks become more brittle and are more prone to instability under external forces. Even coal rocks with good tensile and compressive resistance under normal conditions will deteriorate in terms of the strength and toughness of the rock mass due to changes in the force field and other factors under very deep conditions, resulting in deformation [6]. At the same time, at least 80% of the mines in China have high gas content and poor permeability; low gas permeability pressure, permeability, and saturation; and strong non-homogeneity. In a deep high-pressure environment, coal permeability is seriously insufficient. Many free gas substances are compressed and hidden in coal and rock cavities, accumulating enormous kinetic energy in the coal. In mining and driving, a large amount of gas energy stored in the underground coal seam will instantly burst out when it reaches a certain threshold, resulting in a coal and gas outburst disaster/accident. In general, the risk of coal and gas outburst accidents is directly proportional to the mining depth of a coal mine [7]. After the mine enters the deep mining zone, due to changes in the geological conditions of the coal seam, in situ stress structure, gas stability, and other factors, a mine that has not had coal and gas outbursts or has had soft coal and gas outburst accidents in the shallow mining stage is very likely to have severe coal and gas outburst accidents. Therefore, the risk assessment of coal and gas outbursts in deep coal mines is an essential new topic in this critical field, which has fundamental scientific significance and broad application prospects.

Scholars’ research on coal and gas outbursts mainly involves their mechanism, influencing factors, evaluation, prediction, and risk prevention. Among them, the mechanism and influencing factors of coal and gas outbursts are mainly based on sensitive indicators. These indicators are related to gas adsorption [8], geological structure [9], geological stress [10], gas pressure [11], temperature [12], soft coal seams [13], moisture content [14], porosity [15], gas diffusion [16], deformation [17], the critical value [10], permeability [18], seams [19], etc. The research methods of coal and gas outburst assessment are mainly numerical simulations and modeling, which have mainly focused on the gas management and gas outburst risk assessment of Shimen coal uncovering and the tunneling face [20]. Related research mainly includes traditional evaluation based on subjective and objective weighting and intelligent evaluation based on machine learning.

The traditional evaluation mainly includes subjective and objective weighting methods and the combination of subjective and objective weighting. Standard subjective evaluation methods include questionnaires, expert interviews, expert scoring, analytic hierarchy process, etc. Traditional objective evaluation methods include the entropy weight method, gray theory, rough set theory, fuzzy theory, set pair analysis, extension analysis, etc. Given the advantages and disadvantages of subjective and objective evaluations, the combination of subjective and objective assessments is gradually being recognized. This approach mainly includes the combination of two or more methods, such as hierarchical analysis, gray correlation analysis, distance discriminant analysis, the entropy method, gray target theory, object element analysis, extension evaluation, fuzzy theory, K-means cluster analysis, principal component analysis, accident tree evaluation, and the rough set method [21,22,23,24,25]. With the increasing maturity of computer technology, deep learning, machine learning, and intelligent algorithms have been gradually applied to disaster evaluation and prediction. The commonly used intelligent evaluation algorithms include artificial neural networks [26] (hereafter referred to as ANNs) and support vector machines [27] (hereafter referred to as SVMs). Although ANNs have robust learning and computing abilities and unique advantages in dealing with complex system evaluation, ANNs require a large amount of data, and coal and gas outbursts are typically high-dimensional, nonlinear, small-sample problems. The so-called high dimensionality means that deep coal and gas outbursts are not determined by a single or small number of factors but are the result of the joint action of many factors, such as the physical properties of coal, gas endowment characteristics, mining depth, etc. It is a typical complex brittle system, and the elements do not simply follow the linear development law but present specific nonlinear characteristics. In terms of the simplicity of the object involved, the simpler the problem, the fewer the samples needed to explain it theoretically. Otherwise, a large amount of data is required. When the problem’s simplicity does not correspond to the amount of sample data, and the amount of sample data is small, it is a small sample. A coal and gas outburst is a typical small-probability event, and the related data collection is inherently inadequate. Meanwhile, the high-dimensional characteristics of coal and gas outbursts in deep coal mines further strengthen the small-sample property. Therefore, SVM has a natural advantage in dealing with coal and gas outbursts in deep coal mines. At present, SVM is widely used in the evaluation and prediction of coal and gas outbursts, and relevant scholars have made many improvements on the basis of the traditional SVM, such as adaptive SVM [28], rough set–support vector machine (RS-SVM) [29], rough set–clone selection algorithm–support vector machine (RS-CSA-SVM) [30], complete chaotic particle swarm optimization–support vector machine (CCPSO-SVM) [31], quantum genetic algorithm–least-squares support vector machine (QGA-LSSVM) [32], NN-SVM [33], fruit fly optimization algorithm–support vector machine (FOA- SVM) [34], etc.

Adaptive SVM is a risk prediction model obtained using the adaptive genetic algorithm to improve the time domain exponent a, penalty coefficient C, and kernel function parameter σ of the fuzzy support vector machine, which may be suitable for the complex characteristics of coal and gas outbursts. To reduce the complexity of SVM implementation, RS-SVM was developed, which is a model that reduces the data of coal and gas outburst risk data through a rough set, extracts the core discriminant index, and performs risk discrimination. RS-CSA-SVM is an SVM parameter vector optimized by a clone selection algorithm based on RS-SVM and is an improved algorithm to improve the efficiency and accuracy of the model operation. CCPSO-SVM is a prediction model that uses the multifractal spectrum of the time series of dynamic changes in coal gas emission in front of the mine face as the characteristic index, and it uses the improved completely chaotic particle swarm optimization algorithm and the minimum classification error rate criterion of the test set sample set to select and optimize the SVM parameter vector. QGA-LSSVM is a kind of coal and gas outburst prediction model that uses a quantum genetic algorithm to optimize the parameters of LSSVM, makes full use of the characteristics of the quantum genetic algorithm for parameter optimization, and optimizes the penalty parameter C and kernel parameter σ of LSSVM to improve the prediction accuracy and global search ability of LSSVM. The Drosophila optimization algorithm carries out the global optimization of SVM, and the FOA algorithm finds the optimal combination of each parameter of the support vector machine. The prediction model of FOA-SVM is established to solve the empirical dependence of the setting of each parameter in SVM and the problem of a significant network error to improve the performance of risk prediction. NN-SVM is a discriminant model obtained by pruning the training set and then using SVM to train each sample according to the similarities and differences between each sample and its nearest neighbor. The common feature of the above models is that they entirely rely on the advantages of SVM in solving problems with small samples, high dimensions, and complex nonlinearities and optimize the training parameters of SVM with the help of various intelligent algorithms to save training time and improve training accuracy.

Therefore, the key to studying the risk assessment of coal and gas outbursts in deep coal mines is determining how to solve the complex data-processing problem of coal and gas outbursts in deep coal mines based on the features of deep coal mines. In this paper, according to the characteristics of the small sample, high dimensionality, and nonlinearity of deep coal and gas outbursts, an intelligent algorithm and deep learning are effectively fused, and an improved quantum particle swarm optimization support vector machine (IQPSO-SVM) for deep coal and gas outburst evaluation is proposed. This method is mainly based on the fact that deep coal and gas outbursts are small-probability events and naturally have the characteristics of a small sample. As a commonly used machine learning classification algorithm, SVM has particular advantages in solving small-sample, high-dimension, local-minimum, and nonlinear problems. However, SVM’s classification accuracy is closely related to its built-in penalty parameter C and kernel function parameter g. The traditional SVM must find and set the best parameters and select the appropriate kernel function by experience. The parameter settings significantly influence the generalization ability and accuracy, and it takes considerable time to adjust the parameters. As an optimization algorithm, particle swarm optimization (PSO) is more suitable for global optimization. The particle swarm optimization algorithm embedded in SVM can directly operate the structural objects and optimize the traditional support vector machine parameters to the maximum extent. This algorithm improves the risk prevention and control of coal and gas outbursts in deep coal mines, changes the safety risk from passive risk control to pre-control source prevention, and can effectively improve the safety control of coal mines.

In conclusion, the risk assessment of coal and gas outbursts in deep coal mines has a wide range of practical needs. A coal and gas outburst in a deep coal mine is an extraordinarily complicated and changeable system. The system’s complex and inherent changeable properties greatly aggravate the risk degree of coal and gas outbursts in a deep mine. It also means that even minor vulnerabilities can cause significant coal and gas outbursts. Therefore, when evaluating the risk of coal and gas outbursts in deep coal mines, the possibility of safety accidents should be considered from all angles. Traditional risk assessment methods are difficult to apply to the risk assessment of coal and gas outbursts in deep coal mines due to the lack of information processing ability for risk factors and the complex and changeable factors affecting the risk of coal and gas outbursts in deep coal mines.

## 2. Evaluation Index and Data Source

### 2.1. Evaluation Index of Coal and Gas Outbursts in a Deep Coal Mine

A coal and gas outburst accident is a dynamic failure phenomenon caused by the interrelation between internal stress and gas occurrence characteristics under specific coal and rock conditions [9]. Therefore, a coal and gas outburst is a process caused by energy accumulation, which cannot be separated from the joint action of gas occurrence conditions in coal rocks and coal-body structural characteristics [35]. Based on a literature review, this paper summarizes the main factors affecting deep coal and gas outbursts as follows.

#### 2.1.1. Mining Depth

The depth of mining has an essential influence on coal’s gas content and pressure. With the continuous deep mining of a coal seam, the gas permeability of coal becomes lower and lower, and a large amount of gas is stored in the coal body, increasing the outburst risk of the coal body [36]. There is no unified concept about deep coal mining in academia at present. Generally, underground mining operations with a depth of more than 600 m are called deep mining, mainly because the mining environment changes significantly when the mining is carried out below 600 m. The first is the high ground temperature. With every 100 m increase in the mining depth, the airflow temperature of the mining face increases by about 1.5 °C. High temperature in the deep well has become a common phenomenon and is widely concerning. The second is high ground pressure. The mining depth is proportional to the original rock stress and tectonic stress. The data show that the vertical protolith stress is 20 MPa when the mining depth is 800 m. Under such high pressure and affected by mining disturbance, the internal force of coal is several times or even dozens of times higher than that under normal conditions. A high temperatures and high pressure in deep wells, the brittleness of coal rocks is enhanced. It is easier to destabilize under external forces, resulting in deformation and increasing the risk of dynamic disasters such as coal and gas outbursts. The third is high gas content and low coal permeability. The gas pressure of the coal seam increases in a gradient with the increase in the coal seam burial depth. In a deep high-pressure environment, the gas permeability of the coal body is seriously insufficient, and many free gas substances are compressed and hidden in coal rock holes. The enormous kinetic energy accumulated in the coal body quickly leads to the instantaneous explosion of a large amount of gas energy accumulated in the underground coal seam in the process of deep coal mining and excavation, resulting in coal and gas outburst accidents. Although deep coal and gas outbursts are intuitively closely related to the mining depth, they actually occur because the mining environment significantly changes with the increase in mining depth. In the deep mining environment, nonlinear changes in single vital indexes, such as the coal seam gas pressure, gas content, initial gas release velocity of coal, and coal firmness coefficient, are aggravated, and the degree of interaction between indexes increases, significantly aggravating the risk of deep coal and gas outbursts [37].

#### 2.1.2. The Physical Properties of Coal

The physical properties of coal are also among the indexes used to measure the outburst risk of the coal seam, which mainly include the coal structure type and the coal firmness coefficient. According to the structure of the coal and the structure composition, the physical properties of the surface of the crack, cracks and fractures, and the properties of the tensile and compressive strength of coal, the coal structure type is divided into five major categories: no damage, mild damage, intense damage, crushing damage, and damage resulting in powdered coal [38]. The greater the damage degree of coal, the greater the outburst risk, so the type of coal structure is also known as the coal damage type [39].

The coefficient of the ruggedness of coal is a measure of its mechanical properties, such as tensile and compressive strength, failure resistance, and deformation resistance [40]. Generally, the more complicated and more substantial the coal body, the smaller the seam cracks, the greater the destruction work required, and the higher the ground stress and gas pressure required, the stronger the anti-outburst ability. Therefore, under the joint action of a particular ground stress and gas pressure, the softer the coal seam is, the easier it is to be destroyed first. In addition, the softer the coal seam, the worse the air permeability of the coal, and the more cracks in the coal body, and the internal correlation of cracks is not substantial. In this case, the local pressure is often too enormous, and the risk of outbursts rises [10].

#### 2.1.3. Gas Factors

Gas factors mainly include the gas content, initial gas release velocity, gas pressure, and so on [11]. The gas content includes coal seam gas and coal seam resolvable gas content. As the coal seam’s depth increases, the coal seam’s gas content increases. The gas content of the coal seam is related to gas pressure, the adsorption characteristics of coal, porosity, and temperature [41].

Coal’s initial gas emission velocity is the difference between the gas emission in 45~60 s expressed in mmHg and the gas emission within 0~10 s after gas adsorption at one-atmosphere pressure. It reflects the speed of gas release by coal resolution. Generally, when the standard gas content is the same, the higher the initial gas release velocity, the more serious the coal destruction, and the greater the coal and gas outburst risk [42]. Pressure factors mainly refer to high ground stress and coal seam gas pressure in deep coal mining.

Pressure factors mainly refer to high ground stress and high coal seam gas pressure in deep coal mining. The high ground stress in a deep coal seam leads to higher gas pressure and lower permeability in coal rock. Gas readily gathers in a particular range, resulting in a local pressure imbalance in different locations, which will cause significant damage to the coal body. The gas pressure of a coal seam is the force exerted on the surface of coal and rock at 90 degrees when the gas is sealed in the internal cracks of coal and rock works [43]. The original gas pressure of coal is used to measure the kinetic energy of gas contained in coal rock. In other words, it represents the potential of the coal seam gas inside the mine to be released. From the energy perspective, gas pressure is the leading energy source that triggers an outburst. The higher the gas pressure, the higher the energy and the greater the outburst risk.

Based on the above analysis and according to the Detailed Rules on Prevention and Control of Coal and Gas outbursts implemented by the National Mine Safety Administration on 1 October 2019 and the latest version of the Coal Mine Safety Regulations enforced by the Ministry of Emergency Management of the People’s Republic of China on 1 April 2022, the specific indicators of coal seam outburst risk identification are shown in Table 1. When all indicators align with the conditions listed in Table 1 or when obvious outburst risk factors, such as a jet hole and top drill, occur during drilling construction, it will be identified as an outburst coal seam. Otherwise, the outburst risk of the coal seam should be determined by a comprehensive analysis of the appraisal situation, combined with the actual situation of the original gas content measured directly. However, it is generally identified as a protruding coal seam when f ≤ 0.3 and P ≥ 0.74 MPa; 0.3 < f ≤ 0.5 and P ≥ 1.0 MPa; 0.5 < f ≤ 0.8 and P ≥ 1.50 MPa; or P ≥ 2.0 MPa.

At the same time, when the excavation project is beyond the scope of appraisal, the gas pressure, gas content, and other parameters related to the outburst risk should be measured. The specific regional prediction critical values of coal seam gas pressure and gas content are shown in Table 2.

To sum up, this study assumes that the risk of coal and gas outburst is highly related to the type of coal damage, mining depth, original gas pressure, gas content (including the original gas content and resolvable gas content), the initial gas release velocity of coal, and the robustness coefficient of coal. Thus, the evaluation indexes of deep coal and gas outbursts are established, as shown in Table 3.

### 2.2. The Data Source

Based on the factors above, data acquisition was performed in two deep mines, A and B. Coal mines A and B are located in the alluvial plain of the Huaihe River, with an altitude ranging from +21 to 26 m. The working faces of mines A and B are all below 600 m, both of which are deep mining mines. The mining depth of the working face of mine A is less than 600 m, and that of mine B is less than 900 m. The complexity of the minefield structure was moderate. The data measurement points of coal mine A were concentrated in seams #1 and #2, whereas those of coal mine B were concentrated in coal seams #3 and #4.

Data were obtained based on the combined geological reports of coal mines A and B. The geological reports of coal mines A and B show that the ground stress and coal seam inclination of target mines A and B were generally at the same level (3° to 10°), and the geological structure was moderate. Coal and gas outbursts occurred 38 times in coal mine A, including 8 outbursts, 30 extrusions and tilts, 1 extra-large outburst exceeding 1000 tons, and 7 large outbursts measuring 101–500 tons. Additionally, 7 instances of medium outbursts measuring 50–100 tons and 23 instances of outbursts measuring 50 tons occurred; specifically, these appeared 22 and 2 times in coal seams #1 and #2. The gas weathering zone was located at a depth of −806 m in the elevation of coal seam #1, and the gas content exceeded 8 m^3^/t at a depth of −887 m. The boundary line that distinguishes the absence/presence of outburst hazards in the eastern first mining area of coal seam #2 was located at −882 m, whereas that of coal seam #3 of coal seam B was located at −600 m. Meanwhile, coal seam #4 was located at −695 m.

In this study, the mine and gas outburst risk levels are represented by 1, 2, 3, and 4, where 1 represents a low gas outburst risk (0–5.52 m^3^/t); 2 is a medium gas outburst risk (5.52–12.07 m^3^/t); 3 is a high gas burst risk (12.07–16.92 m^3^/t); and 4 is an extremely high gas burst risk (above 16.92 m^3^/t) [42]. A total of 124 sets were collected; the details are shown in Table 4.

## 3. Evaluation of Coal and Gas Outbursts in Deep Coal Mine Based on IQPSO-SVM

### 3.1. QPSO

The PSO algorithm is a guided intelligent algorithm that imitates bird foraging [44]. It is assumed that m particles form a particle swarm in N-dimensional space: $P=(x_{1},x_{2},\ldots ,x_{i},\ldots x_{m}){}^{T}$; the position and velocity values of particle i are denoted, respectively, as: $x_{i}=\left(x_{i1},x_{i2},\ldots ,x_{id}\right)$ and $V_{i}=\left(v_{i1},v_{i2},\ldots ,v_{id}\right)$, and the particle velocity update formula can be expressed as:

(1) $$v_{id}^{t+1}=\omega \cdot v_{id}^{t}+c_{1}\cdot r_{1}\left(pbest_{id}-x_{id}^{t}\right)+c_{2}\cdot r_{2}\left(gbest_{id}-x_{id}^{t}\right)$$

The position update formula can be expressed as:

(2) $$x_{id}^{t+1}=x_{id}^{t}+v_{id}^{t}$$

In this formula, t represents the current iteration number of the particle; ω is the inertia weight; $x_{id}^{t+1}$ denotes the position of the ith particle at t+1; $v_{id}^{t+1}$ denotes the velocity of the ith particle at time t+1 $\left(i=1,\ldots \;,N;\;N\;\mathrm{is}\;\mathrm{the}\;\mathrm{number}\;\mathrm{of}\;\mathrm{particles};d=1,\ldots \;D;\;D\;is\;\mathrm{the}\;\mathrm{particle}\;\mathrm{dimension}\right)$; $c_{1}$ is the individual acceleration factor; $c_{2}$ is the social acceleration factor; $r_{1}$ and $r_{2}$ are random numbers within $\left[0,1\right]$; $pbest_{id}$ is the individual optimal position; and ${gbest}_{id}$ is the global optimal position. Standard PSO has a maximum velocity limit, and the search range is limited to the vicinity of the current position, which makes it easy to fall into local minima [45,46]. In contrast, quantum particle swarm optimization (QPSO) has a better overall search capability by finding the particle position through the Monte Carlo method using the wave function to represent the particle state, and the particles can be searched throughout the space [47,48].

Particle motion under the quantum concept is used to describe the quantum dynamics equation as:

(3) $$ih\frac{\partial }{\partial t}\varphi \left(x,t\right)=-\frac{h^{2}}{2m}\nabla \varphi \left(x,t\right)+V\left(x\right)\varphi \left(x,t\right)$$

In the formula above, m is the particle mass; h is the Planck constant; and $V\left(x\right)$ is the potential field of the particle.

Assuming that particle p has only one dimension, a one-dimensional δ potential well is established at point p; then:

(4) $$V\left(x\right)=-\gamma \delta \left(x-p\right)=-\gamma \delta \left(y\right),\;y=x-p$$

Then, the stationary Schrodinger equation of the particle in the δ potential well is:

(5) $$\frac{d\varphi }{dy^{2}}+\frac{2m}{h^{2}}\left[E+\gamma \delta \left(y\right)\right]\varphi =0$$

where E is the energy of the particle, and the following can be obtained by solving the equation:

(6) φy=1Lexp−yL,  L=h2mγ

The initial QPSO equation can be simplified as follows:

(7) $$x=p\pm \frac{L}{2}ln\left(\frac{1}{u}\right)$$

where u is a random number with a uniform distribution in the interval $\left(0,1\right)$. The following changes are made to Equation (7):

(8) $$x_{ij}\left(t+1\right)=p_{ij}\left(t\right)\pm \alpha \times \left|g_{Best}-x_{i,j}\left(t\right)\right|\times ln\left[1/u_{i,j}\left(t\right)\right]$$

In the formula above, α is the shrinkage–expansion coefficient, which is the only regulatory variable in the quantum particle swarm, except for the number of particles and the number of runs, and is mainly used to control the speed of particle evolution; $x_{ij}\left(t+1\right)$ is the position of the particle at time t+1; $p_{ij}\left(t\right)$ is point p’s position where the particle appears at time t; $u_{i,j}\left(t\right)$ is a uniformly distributed random number with the interval $\left(0,1\right)$; $x_{i,j}\left(t\right)$ is the position of the particle at time t; and $g_{Best}$ is the average of the individual optimal values of all particles. Once α is determined, the QPSO evolution equation only includes the position to describe the particle, so the QPSO algorithm only evaluates the potential well by the average optimal position. Therefore, the model is more concise, and the training cooperation ability and the overall search function are more vital.

The center position of the *i*th particle potential well is:

(9) $$p=\frac{r_{1}\left(t\right)\times p_{i}+r_{2}\left(t\right)\times p_{g}}{r_{1}\left(t\right)+r_{2}\left(t\right)}$$

The expression of $g_{Best}$ is:

(10) $$\begin{array}{c} g_{Best}=\frac{1}{n}\sum_{i=1}^{n}p_{i}\left(t\right)=\\ \left[\frac{1}{n}\sum_{i=1}^{n}p_{i1}\left(t\right),\frac{1}{n}\sum_{i=1}^{n}p_{i2}\left(t\right),\cdots ,\frac{1}{n}\sum_{i=1}^{n}p_{iD}\left(t\right)\right] \end{array}$$

Generally, the value of α is controlled by the linear reduction method, which is as follows:

(11) $$\alpha =\alpha_{min}+\left(\alpha_{max}-\alpha_{min}\right)\frac{t_{max}-t}{t_{max}}$$

In the formula, the larger the value of α, the faster the global convergence, but it affects the accuracy of the model, and vice versa for the local search, but it takes longer. $\alpha_{max}$ is the initial value of the contraction–expansion coefficient, and $\alpha_{min}$ denotes the final value. According to experience, generally, $\alpha_{max}=1$ and $\;\alpha_{min}$ = 0.5. From Formula (11), the value of α linearly decreases. As the number of iterations increases and reaches a specific value, the optimal position of a single particle will become increasingly close to the optimal part of the population, and the population diversity will decrease. Therefore, it is difficult for the linearly decreasing α to meet the practical needs of dynamic changes, and QPSO still has the problem of premature convergence. To obtain more appropriate parameters for particles in the optimization process, this paper introduces the improved quantum particle swarm optimization (IQPSO) algorithm [49,50].

### 3.2. IQPSO

(1) Average optimal location improvement

The QPSO algorithm changes with the particle position, and the particle fitness changes as a whole and determines the population state based on it. Suppose that M is the number of particles in the population, the current fitness of particle i is $f_{i}$*,* the average fitness of the population is $\bar{f}$, and the fitness variance $s^{2}$ is:

(12) $$s^{2}=\sum_{i=1}^{M}{\left(f_{i}-\bar{f}\right)}^{2}\;$$

At this time, $s^{2}$ reflects the aggregation degree of particles in the whole population. The smaller $s^{2}$ is, the more compact the particles are. On the contrary, the more dispersed the particle aggregation, the smaller Q gradually becomes as the algorithm iterates. Therefore, the particles will become increasingly compact in the later stage. The algorithm will be premature when $s^{2\;}$ is small relative to the threshold σ. To prevent the algorithm from falling into the above situation, the evolution factor $\lambda_{t}$ is added when the average optimal position is obtained:

(13) $$\lambda_{t}=\mu_{1}\left[K_{t}\left(0,1\right)+\mu_{2}N_{t}\left(0,1\right)\right]$$

In the above equation, $K_{t}\left(0,1\right)$ is any number within the interval $\left(0,1\right)$ generated by the Cauchy distribution, $N_{t}\left(0,1\right)$ is any number within the interval $\left(0,1\right)$ produced by the Gaussian distribution, and $\mu_{1\;}$ and $\mu_{2}$ are interference coefficients. Its expression is as follows:

(14) $$\left\{\begin{array}{c} \mu_{1}=\mu_{1min}+\left(\mu_{1max}-\mu_{1min}\right)\frac{t}{t_{max}}\\ \mu_{2}=\mu_{2max}+\left(\mu_{2max}-\mu_{2min}\right)\frac{t}{t_{max}} \end{array}\right.$$

In the above formula, $\mu_{1min}$ and $\mu_{1max}$ respectively represent the minimum and maximum values of $\mu_{1}$; $\mu_{2min}$ and $\mu_{2max}$ are the minimum and maximum values of $\mu_{2}$. t represents the current iteration number, and $t_{max}$ represents the maximum iteration number. After increasing the evolution factor, the average ideal position $\bar{C\left(t\right)}$ is expressed as:

(15) $$\bar{C\left(t\right)}=\lambda_{t}\cdot C\left(t\right)$$

$\mu_{1}$ is a linearly increasing process. A small value at the beginning of the iteration benefits local convergence, while $\mu_{1}$ is beneficial for the global search as the operation gradually deepens. The change in $\mu_{2}$ can make the search range wider to improve the algorithm.

(2) Improvement of shrinkage–expansion coefficient α

The traditional α coefficient update formula is a linear decreasing line, which makes it challenging to entirely reflect the actual dynamic changes of the model. Therefore, according to the idea of dynamic parameter changes in the PSO algorithm, this paper adopts an adaptive nonlinear change method to control the parameter, and the specific update method is as follows:

(16) $$\alpha_{2}=\alpha_{max}-\left(\alpha_{max}-\alpha_{min}\right)tan\left(\frac{t}{t_{max}}\cdot \frac{\pi }{4}\right)$$

**Figure 1:**
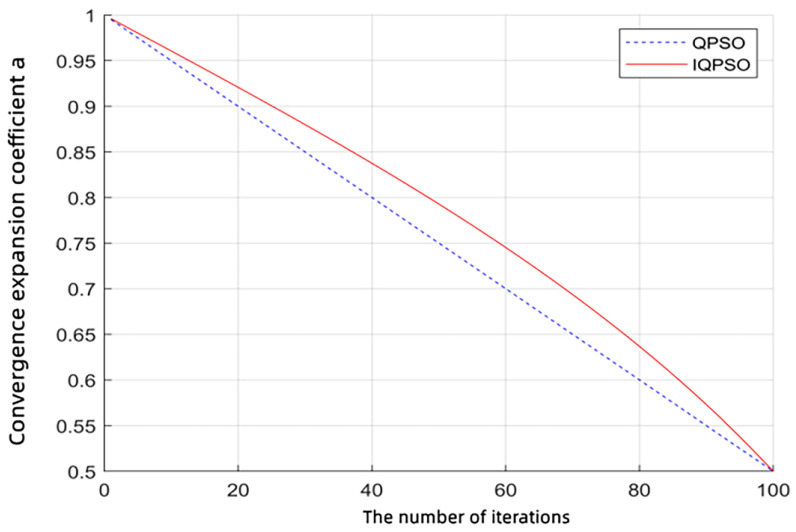
Comparison of the change in the shrinkage–expansion coefficient a for the linear decreasing strategy and the modified decreasing strategy.

$\alpha_{max}$ and $\alpha_{min}$ represent the initial and final values of the shrinkage–expansion coefficient, respectively, which are generally set as $\alpha_{max}$ = 1 and $\alpha_{min}$ = 0.5, according to experience. Figure 1 shows the changes in the shrinkage–expansion coefficient a using the traditional linear decreasing strategy and the improved decreasing strategy when there is no difference in any other parameters and conditions and the maximum number of times is 100. In the figure above, at the initial stage of particle evolution iteration, the value of the improved decreasing strategy (solid red line in the figure) is more significant than that of the updated process with the traditional a value (dashed blue line), which is more conducive to the global search of the algorithm. At a later stage of the iteration, the value obtained with the improved algorithm’s decreasing strategy gradually becomes more diminutive than that using the traditional one. The former decreases faster than the latter, which is conducive to strengthening the local search. The iteration speed of IQPSO and QPSO is reflected by the time consumed to optimize SVM. The influencing factors of the iteration speed include the number of particle swarms, evolutionary algebra, the individual acceleration factor, the social acceleration factor and the data set size. In this study, 74 of 124 data sets were randomly selected as the training set, and the remaining 50 sets were used as the test set. Under the condition that the individual acceleration factor *C*1 was 1.5, the social acceleration factor *C*2 was 0.7, the population number was 50, and the evolutionary algebra value was 200, the QPSO-SVM and IQPSO-SVM were both 1.5. The operation time was 7.8610 s and 9.2362 s using the optimal penalty coefficient and kernel function parameters, respectively (see Table 5), which proves that the iteration speed of IQPSO is indeed better than that of QPSO. It can be seen that the improved decreased updating strategy can significantly improve the global search and local search abilities of the algorithm, regardless of which stage is before and after.

### 3.3. IQPSO-SVM

**Figure 2:**
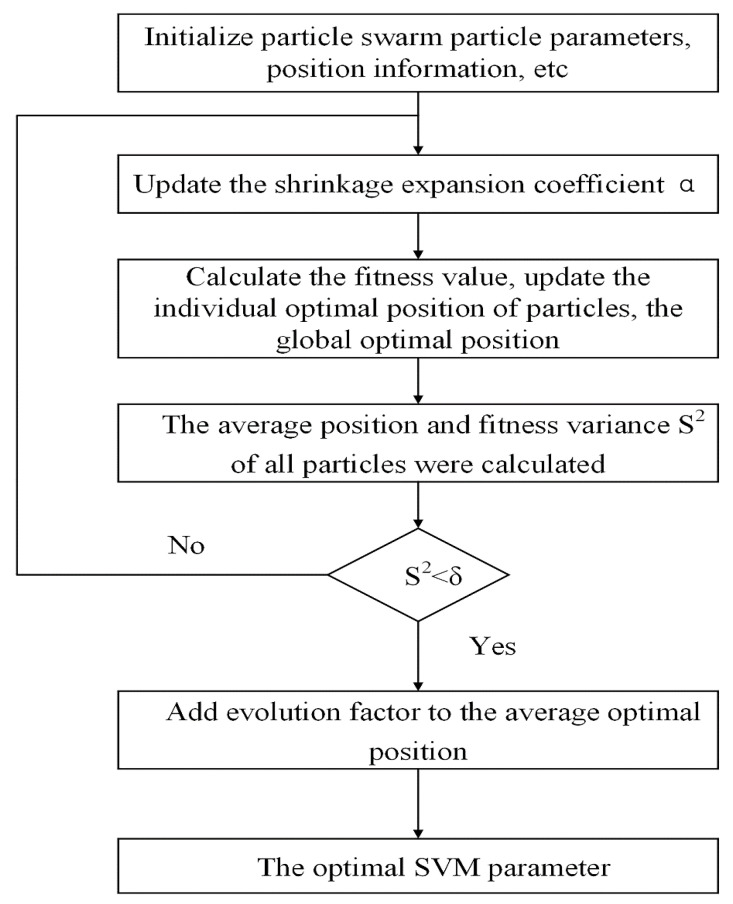
Flowchart of IQPSO-SVM algorithm.

According to the advantages of the SVM classification algorithm in solving small-sample, high-dimensional, and nonlinear problems [51], this paper proposes an improved quantum particle swarm optimization support vector machine (IQPSO-SVM) method for deep coal and gas outburst evaluation. IQPSO-SVM was chosen over the standard SVM because the latter’s classification accuracy and generalization ability are affected by the choice of the penalty parameter (C) and kernel function parameter (g), and it takes considerable time to adjust these parameters [52]. As an optimization algorithm, the PSO algorithm has good global optimization ability, and embedding it into SVM can optimize the parameters of the support vector machine to the maximum extent [53,54]. However, the reliability of the PSO algorithm results is closely related to the restriction degree of the connection between particles. At the beginning of the operation, particles will continue to converge to an ideal position along a certain path at a fast speed. However, when the process reaches a certain degree, the particle movement speed begins to weaken and grows infinitely close to zero. At this time, the particle population loses the ability to evolve further, and “convergence” stops or “converges” prematurely. This situation arises because the homogeneity is too strong, and the difference is insufficient among particles. In this case, particles will be limited to a specific range and cannot find the optimum in the whole field. The successful discovery of new particles is needed to break this limitation. Based on this, IQPSO-SVM is proposed in this paper. The specific IQPSO-SVM algorithm process is shown in Figure 2.

## 4. Empirical Analysis and Results Analysis

### 4.1. SVM Kernel Function Selection

Currently, the following SVM kernel functions are commonly used:

Linear kernel function:

(17) $$K\left(x,x_{i}\right)=\left(x,x_{i}\right)$$

Polynomial kernel function:

(18) $$K\left(x,x_{i}\right)={\left[\left(x.x_{i}\right)+1\right]}^{q}$$

Radial basis function (RBF):

(19) $$K\left(x,x\_i\right)=exp\left\{-\frac{{\left|x-x_{i}\right|}^{2}}{\sigma^{2}}\right\}$$

Sigmoid kernel function:

(20) $$K\left(x,x_{i}\right)=tanh\left[v\left(x,x_{i}\right)+c\right]$$

A total of 124 groups of data were selected in this study. According to the above, coal and gas outbursts were classified into four grades, among which 39 groups of coal and gas outbursts were grade 1, 28 groups of coal and gas outbursts were grade 2, 32 groups of coal and gas outbursts were grade 3, and 25 groups of coal and gas outbursts were grade 4. The RAND function in MATLAB was used to randomly select 74 groups of data as the training set, and the remaining 50 groups of data were used as the test set. There were 74 groups of data in the statistical training set, and 24, 17, 18, and 15 groups of data were in grades 1, 2, 3, and 4, respectively; there were 50 groups of data in the test set, and 15, 11, 14, and 10 groups of data were in grades 1, 2, 3, and 4, respectively. For both the training and test sets, the randomly selected data were more uniform, and the mutual verification results were more credible. Seventy-four groups of randomly selected training set data and fifty groups of test set data were substituted into the SVM model with the above four different kernel functions for model training and comparative testing (Table 6).

**Figure 3:**
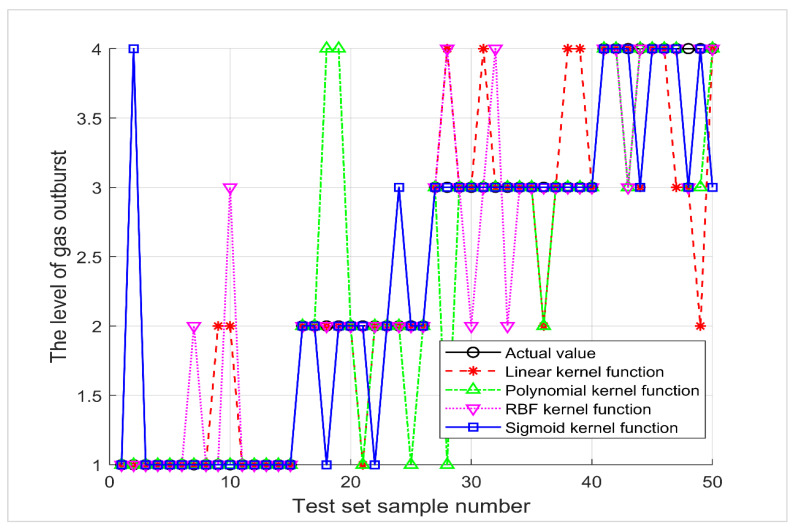
Comparison of four kernel function fits: linear, polynomial, radial basis, and Sigmoid.

The linear kernel function correctly classified 38 samples out of 50 test data sets, with a correct discriminant rate of 76%. The polynomial kernel function accurately discriminated 41 models, with a correct discrimination rate of 82%. The radial basis (RBF) kernel function correctly discriminated 42 samples, with a correct rate of 84%. The Sigmoid kernel function determined 43 samples correctly, with an accuracy rate of 86%. Comparing the classification results of the above four kernel functions (Figure 3), the Sigmoid kernel function is the best in this numerical fitting, so the Sigmoid kernel function was used by default in the following analysis, and this result was used as the output of the standard SVM.

### 4.2. IQPSO-SVM Results Analysis

**Figure 4:**
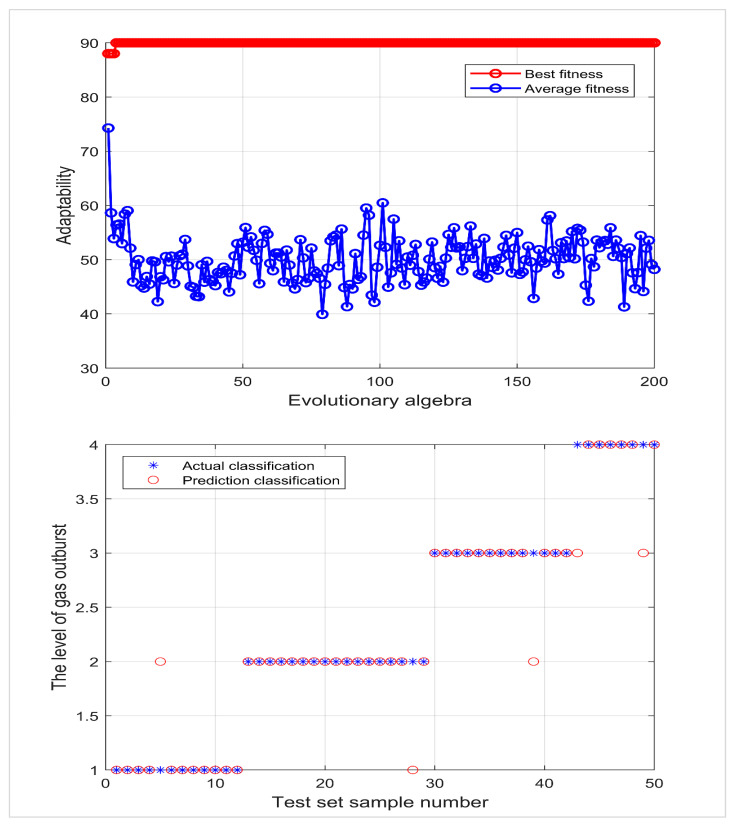
PSO-SVM fitness curve and actual classification results.

**Figure 5:**
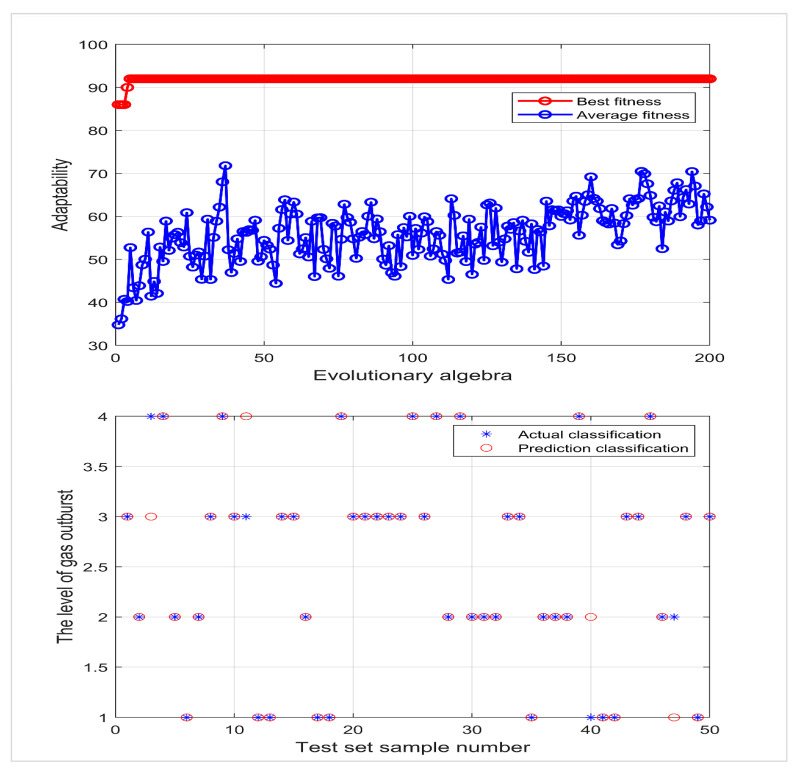
QPSO-SVM fitness curve and actual classification results.

**Figure 6:**
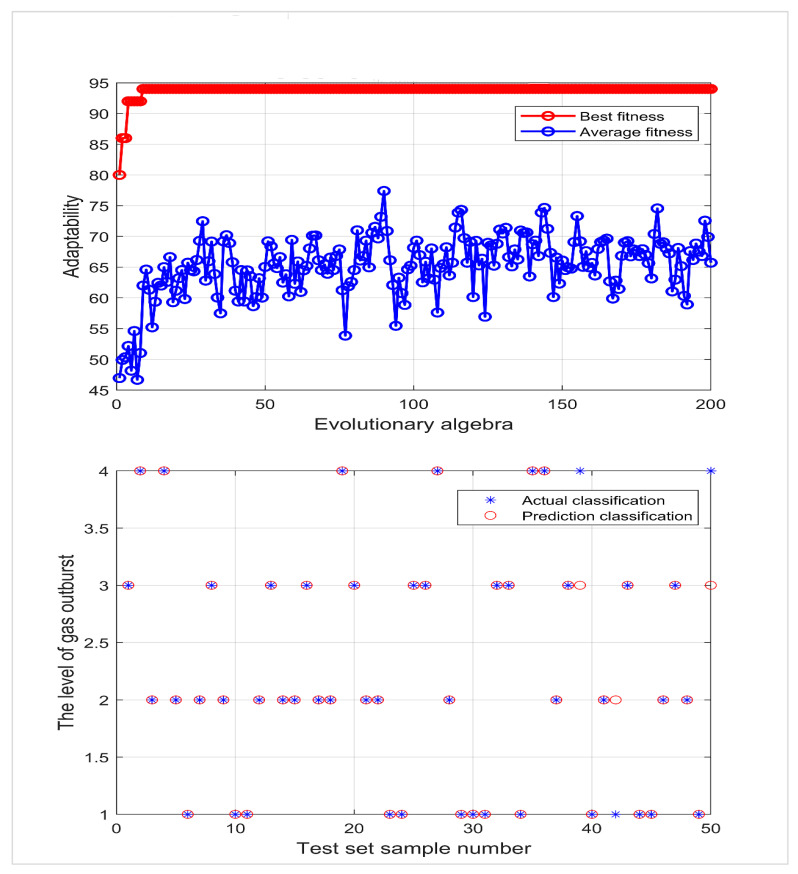
IQPSO-SVM fitness curve and actual classification results.

The Sigmoid function was selected as the SVM kernel function, with seven risk evaluation indexes as the input and the gas outburst risk level as the output; 74 groups of data were randomly selected from 124 groups of data as the training set, and 50 groups of data were used as the test set, which were respectively input into PSO-SVM, QPSO-SVM, and IQPSO-SVM for comparative testing. The parameters of the PSO algorithm were set as follows: the maximum number of runs was 200 times, the overall population number was 50, C1=1.5, C2=1.7, and the inertia connection weight was set as 1. See Figure 4, Figure 5 and Figure 6 for the run results.

Figure 4 shows that when the optimal penalty coefficient of PSO-SVM is 12.7545 and the optimal kernel function parameter is 0.62683, 45 of 50 groups of test data are correctly discriminated, and the discrimination accuracy is 90%.

Figure 5 shows that when the optimal penalty coefficient of QPSO-SVM is 220.8754 and the optimal kernel function parameter is 10.2332, 46 of 50 groups of test data are correctly discriminated, and the discrimination accuracy is 92%.

Figure 6 shows the support vector machine optimized by the improved quantum particle swarm optimization algorithm. When the optimal penalty coefficient is 66.0565 and the optimal kernel function parameter is 0.30788, 47 of 50 groups of test data are correctly discriminated, and the discrimination accuracy is 94%.

It can be seen that IQPSO-SVM significantly improves the generalization ability of the results and shows a high classification accuracy rate in this simulation. The complete test comparison results for evaluating deep coal and gas outbursts are IQPSO-SVM > QPSO-SVM > PSO-SVM > standard SVM, and the specific comparison results are shown in Table 5.

## 5. Discussion

According to the objective reality of deep mining in Chinese coal mines, this study adopted an appropriate mathematical transformation and reasonable modeling to judge the risk degree of deep coal and gas outbursts. A deep coal and gas outburst is a typical small-sample, high-dimensional, and complex nonlinear problem. The premise of the accurate evaluation of deep coal and gas outbursts is to capture the nonlinear variation law of deep coal and gas outbursts by proper methods. Based on balancing the calculation time and detection accuracy, this paper proposes a risk assessment method for deep coal and gas outbursts based on IQPSO-SVM.

First, in the evaluation accuracy, an essential basis that affects the model training accuracy is the characteristics of the sample data. In machine learning, neural networks have advantages in dealing with significant sample size problems. However, a deep coal and gas outburst is a small-probability event and naturally has the characteristics of a small sample. The total number of samples collected in this paper is 124 groups, a small sample of data. SVM has particular advantages in solving small-sample, high-dimension, local-minimum, and nonlinear problems. Moreover, the SVM algorithm is simple and robust and has good generalization ability. However, SVM’s classification accuracy is closely related to its built-in penalty parameter C and kernel function parameter g. Therefore, based on the correct selection of the kernel function, this study used IQPSO to optimize the penalty parameter C and kernel parameter g of SVM to balance the global search and local search problems in the algorithm design. The data fitting shows that IQPSO can combine the unique advantages of SVM in solving small-sample, high-dimension, local-minimum, and nonlinear problems. The penalty parameter C and kernel parameter G of SVM are optimized, and the parallelism, stability, robustness, global optimality, and model generalization ability of data fitting are improved. Compared with the test results of the standard SVM, PSO-SVM, and QPSO-SVM models, IQPSO-SVM constructs a more reasonable adaptive recognition model that can be used in the field of gas outburst recognition in deep coal mines. This model can significantly improve the detection accuracy of deep coal and gas outbursts, and the accuracy of the target identification of coal mine disasters is increased to 94%.

Secondly, in terms of training time, the traditional SVM takes considerable time to adjust the parameters, while PSO, as an optimization algorithm, has better global optimization ability. By embedding the PSO algorithm into SVM, the object structure can be operated directly, and the parameters of the traditional support vector machine can be optimized to the maximum extent. However, the reliability of the PSO algorithm is closely related to the restriction degree of the relationship between particles. In solving many high-dimensional and nonlinear problems, the PSO algorithm is limited to a specific search range due to the substantial homogeneity of particles and the insufficient difference between particles. It cannot find the ideal value in the whole range. This situation is called convergence stop or premature convergence. Based on the above understanding, this study constructed the IQPSO-SVM evaluation method for deep coal and gas outbursts through an appropriate transformation. The problems of optimal local risk and premature convergence in the training process of PSO and QPSO are effectively solved, and the learning ability of SVM and the global search function of the IQPSO algorithm are used. The data fitting shows that PSO-SVM > QPSO-SVM > IQPSO-SVM in terms of overall time consumption. Therefore, IQPSO-SVM maintains a high detection accuracy, saves time, and is more efficient in evaluating deep coal and gas outbursts.

Finally, the IQPSO-SVM method proposed in this paper comprehensively uses the knowledge and research tools of management science, security science, computer science, security engineering, and other fields. Real-time data are integrated with the intelligent algorithm for machine learning classification processing, and the risk level is determined. It provides a method for improving the early warning system of deep coal and gas outbursts and carrying out the hierarchical prevention and control management of deep coal and gas outbursts. For the scientific evaluation of deep coal and gas outburst risk, this method promotes deep coal mine safety management and provides a new train of thought on deep coal mine safety, but it can also be extended to other areas, such as machine-learning-integrated intelligent algorithms of data processing methods to solve other complex small-sample, high-dimensional, nonlinear problems, and provides an essential reference. However, the difference between theory and practice is a specific problem situation. The problem situation determines the input and output settings, the problem’s index and dimension, the sample size requirement, and the training model, directly affecting the evaluation results. Therefore, the evaluation method of machine learning classification processing based on data fusion by the intelligent algorithm proposed in this paper enriches the theoretical method of coal mine safety evaluation and shifts the safety risk evaluation of deep coal mines from theory to the broader application level.

## 6. Conclusions

### 6.1. A Set of Crucial Index Evaluation Systems of Coal and Gas Outbursts in the Deep Coal Mines Is Established

This paper’s key contribution is establishing an index of deep coal and gas outbursts. Deep coal and gas outbursts are closely related to the mining depth. Significant changes occur in the deep mining environment, typically characterized by three high parameters and one low one, namely, high ground temperature, high ground pressure, high gas content, and the low permeability of coal and rock. In this environment, the gas permeability of the coal body is insufficient, and the influence of mining disturbance enhances the brittleness of coal and rock. The internal force of coal rock is much higher than usual, and it is more likely to become unstable under the action of an external force. More importantly, in the deep environment, the nonlinear changes in single vital indexes, such as the coal seam gas pressure, gas content, initial gas release velocity of coal, and coal firmness coefficient, are aggravated, and the degree of interaction between each index is increased, significantly aggravating the risk of deep coal and gas outbursts. Based on the above understanding, in this paper, based on a literature review, combined with guiding documents such as Rules for Prevention and Control of Coal and Gas Outburst and Coal Mine Safety Regulations, the type of coal damage, mining depth, original gas pressure, gas content (including original gas content and resolvable gas content), the initial gas release velocity of coal, and the ruggedness coefficient of coal are established as a critical index system of deep coal and gas outbursts.

### 6.2. IQPSO-SVM Model Based on Small-Sample Features Is Constructed According to the Data Characteristics of Deep Coal and Gas Outbursts

The premise of the accurate evaluation of deep coal and gas outbursts is to capture the nonlinear variation law of deep coal and gas outbursts by proper methods. At the same time, a deep coal and gas outburst is a small-probability event, which naturally has the characteristics of a small sample. SVM has unique advantages in solving small-sample, high-dimension, local-minimum, and nonlinear problems. Based on the comprehensive consideration of the calculation time and detection accuracy, in this paper, using the IQPSO-SVM risk assessment method for deep coal and gas outbursts, combined with the appropriate transformation, the gas outburst index of a deep coal mine is transformed into a specific problem suitable for the IQPSO-SVM algorithm model. Based on the correct selection of the kernel function, improved quantum particle swarm optimization (IQPSO) is used to optimize the penalty parameter (C) and kernel parameter (g) of SVM to balance the global search and local search problems in the algorithm design. The research shows that the detection accuracy of the methods is: standard SVM < PSO-SVM < QPSO-SVM < IQPSO-SVM; in terms of overall time consumption, PSO-SVM > QPSO-SVM > IQPSO-SVM. Therefore, IQPSO-SVM has an excellent performance in saving time, increasing accuracy, and improving efficiency in evaluating the risk of deep coal and gas outbursts.

### 6.3. A Research Idea for Solving Complex Nonlinear Problems Is Provided

According to the data characteristics of the safety evaluation system of gas outburst risk in the deep coal mine, this paper explores and proposes a set of intelligent evaluation methods based on improving the traditional standardized data processing methods. This method can not only deal with uncertain information but also provide the quantitative risk probability value, effectively solving the safety risk assessment of coal and gas outbursts in deep coal mines. However, a deep coal and gas outburst is one of many disaster risks, such as heat damage, water damage, and rock bursts, in the deep coal mine. Complex accident causation theory shows that many aspects increase the safety risk of the deep coal mine. Its causes are complex and interrelated, each kind of disaster affects the other, and the evolution process of the accident is dynamic and changeable. Therefore, a deep coal mine is a complex giant system involving secondary characteristics, derivation, sudden changes, crossover, variability, nonlinearity, fuzziness, strong coupling, and so on. It is a complex system under the joint action of multiple subjects, factors, scales, attributes, levels, and departments. It entails the integration and penetration of cross-level, cross-disciplinary edge-crossing comprehensive problems. The data for this problem are typically complex, highly dimensional, and nonlinear. Machine learning and intelligent algorithms can serve as essential resources for solving this problem.

### 6.4. It Opens up a New Possibility for Promoting the Classification Management of Coal Mine Safety and Improving the Early Warning System of Coal Mine Safety

At present, shallowly buried coal seams are being gradually mined. Deep mining is urgently needed in China’s energy structure and coal mine safety management practice. The deep coal seam faces the complicated objective reality of high ground temperature, high ground pressure, high gas content, and low coal permeability in the mining process, which leads to the significantly increased risk of deep coal and gas outbursts. In this paper, intelligent algorithms and deep learning are effectively integrated. The evaluation of coal and gas outbursts in the deep coal mine is transformed into a model suitable for a specific intelligent optimization algorithm. In the training of the deep learning model, the dialectical relationship between the global search and local search is fully considered to comprehensively improve the intelligence level of data processing and the evaluation accuracy and generalization ability of the evaluation model. A safety assessment method for gas outbursts, closely combined with the situation of the deep coal mine, is proposed. This method opens up a new possibility for promoting deep coal mines’ hierarchical prevention and control management and improving the safety early warning system.

## 7. Contributions and Limitations

According to the current situation of China’s energy structure and coal and mine safety management practice, this paper effectively combines sample data processing, data normalization method improvement, complex evaluation algorithm optimization, and other aspects with scientific and reasonable system design. It not only promotes a new way of thinking for the scientific evaluation of deep coal and gas outburst risk and deep coal and mine safety management but also provides an essential reference for the scientific evaluation of high-dimension, nonlinear problems in other fields. However, between theory and practice is a specific problem situation. The problem situation determines the input and output settings, the problem’s index and dimension, sample size requirements, and training models. As such, the choice of deep coal and gas outbursts involves the thermal damage in the deep coal mine, the damage and impact ground pressure, and so on. The safety risk of a deep coal mine is a disaster risk with many types of complex accident causes. Influenced by complex, interrelated, and mutual influences in the mined-out area and the changeable dynamic evolution process of the accident, the cause is a complex giant system involving secondary characteristics, derivation, sudden changes, crossover, variability, nonlinearity, fuzziness, strong coupling, and so on. Furthermore, it is under the joint action of multiple disciplines, factors, scales, attributes, levels, and departments, entailing the integration and penetration of cross-level, interdisciplinary edge-crossing comprehensive problems. In the future, the safety risk assessment of multiple disasters in deep coal mines should be studied in combination with the specific situation of deep coal mines, especially the coupling evaluation of numerous disasters in deep coal mines in complex environments. Based on a thorough grasp of the problem situation, it can gradually be extended to other high-dimensional and nonlinear problems to expand its application value.

## Figures and Tables

**Table 1:** Coal seam outburst risk identification index.

Discriminant Indicators	Original Gas Pressure (P)	Ruggedness Coefficient of Coal (f)	Destruction Type of Coal	Initial Gas Release Velocity of Coal (ΔP)
The critical value and range of outburst risk	P ≥ 0.74	f ≤ 0.5	Ⅲ, Ⅳ, Ⅴ	ΔP ≥ 10

**Table 2:** Critical values of regional prediction based on coal seam gas pressure and gas content.

Gas Pressure (P/MPa)	Gas Content (W)	Regional Category
P < 0.74	W < 8 (Tectonic belt W < 6)	No outburst danger zone
Other than the above		Danger zone of outburst

**Table 3:** Initial evaluation indexes of deep coal and gas outbursts.

Indicators	Explanation of Indicators	Type of Indicator
Coal damage type $X_{1}$	The degree of coal damage can be divided into 5 grades: undamaged, mildly damaged, generally damaged, intensely damaged, and destroyed into powder. The higher the grade, the greater the risk of gas outbursts.	+
Mining depth (m) $X_{2}$	This generally refers to the vertical height of the mining coal seam from the ground. The deeper the coal seam, the greater the risk of gas outbursts.	+
Original gas pressure (MPa) $X_{3}$	When the coal seam is buried at a certain depth, the stress is caused by the gas in the pores and fissures of the coal seam on the seam wall. The higher the gas pressure, the greater the windiness of gas outbursts.	+
Original gas content (m^3^/t) $X_{4}$	The gas volume of coal per unit weight reflects the gas potential of the coal seam. The higher the content, the greater the potential and the greater the risk of gas outbursts.	+
Resolvable gas content $X_{5}$	The amount of gas released from coal under normal conditions. The greater the amount of precipitation, the greater the risk of gas outbursts.	+
Initial gas release velocity of coal (ΔP) $X_{6}$	It refers to the rate of coalbed methane emission when the coal is initially exposed. It is an index indicating the outburst risk of the coal seam. The greater the initial rate of gas release, the greater the outburst risk.	+
Coal ruggedness coefficient (f) $X_{7}$	This measures the degree of coal rock sturdiness of an index. The more complex the coal seam, the stronger its sturdiness coefficient in its ability to resist outbursts, and the lower the risk of coal and gas outbursts.	−

**Table 4:** Raw data of gas outburst samples from deep mines.

Serial Number	$X_{1}$	$X_{2}$	$X_{3}$	$X_{4}$	$X_{5}$	$X_{6}$	$X_{7}$	Outburst Risk Level
1	2	−912.6	0.84	5.6394	4.5607	6.2	0.9	2
2	2	−898.45	0.84	5.7168	4.6381	6.2	0.9	2
3	5	−873.8	3.5	9.7688	8.8762	5.1	0.9	4
4	2	−870	0.15	1.44	0.17	2.2	0.9	1
5	5	−867	1.56	9.1476	8.0821	7.9	1	3
6	5	−865	2.91	6.4072	5.4442	12.1	0.6	4
7	5	−862	2.35	9.95	8.86	7.5	1.3	4
8	5	−860.1	2.1	7.7383	6.863	10	1	4
9	5	−856	3.18	8.3513	7.6419	7.9	0.7	4
10	5	−851	2.15	8.8312	7.8682	9.6	0.7	4
…	…	…	…	…	…	…	…	…
113	2	−783.4	0.125	1.6692	0.9184	5	1	1
114	2	-760	1.7	4.9096	4.0569	3.6	0.9	3
115	2	−756.2	0.6	3.5352	2.3791	3.3	0.8	2
116	3	−748	1	4.3	3.34	4.5	0.5	4
117	2	−703	0.48	3.2293	2.5231	3.3	1	1
118	3	−703	1.05	5.7075	4.8763	3.4	0.8	3
119	2	−667.5	0.52	5.625	4.8311	3.6	1.2	2
120	2	−650	0.432	3.0898	2.3381	2.7	0.7	1
121	3	−647.5	1.05	5.7075	4.8763	3.4	0.8	3
122	3	−635	1.04	4.4807	3.6564	4	0.9	3
123	2	−630.7	0.487	2.9697	2.2473	4.8	0.7	1
124	2	−627.4	0.519	4.012	3.1706	6.3	0.8	2

**Table 5:** Parameters and prediction results of each training model.

Result	Standard SVM	PSO-SVM	QPSO-SVM	IQPSO-SVM
Individual acceleration factor *C*1	/	1.5	1.5	1.5
Social acceleration factor *C*2	/	1.7	1.7	1.7
Evolution algebra	/	200	200	200
Penalty coefficient (C)	419.02	12.7545	220.8754	66.0506
Kernel function parameter (g)	0.17	0.62683	10.2332	0.30788
Test sample size	50	50	50	50
Correct predictions	43	45	46	47
Prediction accuracy	86%	90%	92%	94%
Time consumed (s)	1.3254	11.6552	9.3262	7.8610

**Table 6:** Classification of different kernel functions.

Kernel Function Category	Accuracy	Svmtrain Parameters
Linear kernel function	76%	‘−c 419.02 −g 0.17 −t 0’
Polynomial kernel function	82%	‘−c 419.02 −g 0.17 −t 1’
Radial basis function (RBF)	84%	‘−c 419.02 −g 0.17 −t 2’
Sigmoid kernel function	86%	‘−c 419.02 −g 0.17 −t 3’

## Data Availability

Not applicable.
